# Innate allorecognition induces HSP70 in monocytes contributing to renal allograft rejection

**DOI:** 10.1172/jci.insight.197348

**Published:** 2026-10-08

**Authors:** Zeping Gui, Neda Feizi, Berkay Demirors, Canxiang Lin, Hehua Dai, Mouhamad Al Moussawy, Andrew Friday, Steven M. Sanders, Latha Halesha, Amanda L. Williams, Faruk Sacirbegovic, Parmjeet S. Randhawa, Khodor I. Abou-Daya, Martin H. Oberbarnscheidt

**Affiliations:** 1 Thomas E. Starzl Transplantation Institute, Department of Surgery, University of Pittsburgh School of Medicine and University of Pittsburgh Medical Center, Pittsburgh, Pennsylvania, USA.; 2 Division of Transplant Pathology, Department of Pathology, University of Pittsburgh School of Medicine, Pittsburgh, Pennsylvania, USA.; 3 Department of Immunology, University of Pittsburgh, Pittsburgh, Pennsylvania, USA.

**Keywords:** Immunology, Nephrology, Innate immunity, Monocytes, Organ transplantation

## Abstract

After encountering allogeneic nonself, monocytes differentiate to DCs, which in turn activate the adaptive immune system that drives transplant rejection. The downstream mechanisms of allorecognition are largely unknown. We analyzed scRNA-seq data sets to identify transcriptional changes in monocytes occurring after allostimulation. *Hspa1a*, which encodes HSP70, was upregulated in monocytes after allostimulation in contrast to syngeneic controls in mice lacking T, B, and NK cells. Similar findings were seen in scRNA-seq data derived from kidney biopsies of rejecting transplant patients. To validate the role of HSP70 in innate allorecognition and transplantation, we performed allogeneic bone marrow plug and kidney transplantation into WT, *Hspa1a/Hspa1b^–/–^*, and DSG-treated (HSP70 inhibitor) B6 mice and examined the graft immune infiltrate. Histology, T cell infiltration, and survival were assessed in kidney transplanted mice. In both models, DSG-treated or HSP70-KO recipients displayed significantly reduced infiltration by monocyte-derived DCs (mo-DC). Chronic rejection of Balb/c kidney grafts in WT B6 recipients was attenuated in DSG-treated and HSP70-KO recipients as indicated by a reduction in Banff score. Further experiments demonstrated that in vitro allostimulated immature HSP70-KO BMDC showed less maturation compared with WT BMDC. Targeting HSP70 in innate immune cells offers a novel approach to reduce chronic kidney graft rejection.

## Abstract 2

In this study we investigate downstream mechanisms of innate allorecognition by monocytes in the context of transplantation, identifiying HSP70 as a key pathway that can be targeted to improve chronic allograft rejection.

## Introduction

Kidney transplant outcomes have only slightly improved over the past few decades (1). Antirejection treatments have focused on nonspecifically suppressing the innate and adaptive immune system primarily by targeting T cells. After transplantation, activation of the adaptive immune system is reliant on cognate antigen presented by innate cells, including monocyte-derived DCs (mo-DC) (2, 3). We have previously shown that monocytes recognize allogeneic nonself; differentiate to mo-DC, which infiltrate the graft; and sustain graft resident T cells that drive kidney allograft rejection (4–6).

In the last few years, several scRNA-seq datasets have been generated by our group and others that included myeloid cells, specifically monocytes and mo-DC, both from transplanted mice and humans from different tissues. This offered an opportunity to mine these datasets for transcriptional changes in monocytes after allogeneic transplantation that are associated with mo-DC differentiation.

In this study, we first analyzed scRNA-seq datasets generated in mice. We found allospecific upregulation of Heat Shock Protein Family A Member 1A (*Hspa1a*), which encodes the inducible form of heat shock protein 70 kDa (HSP70) (7) in monocytes. *HSPA1A* upregulation in graft infiltrating monocytes was also observed in kidney graft biopsies of patients with rejection. HSP70-deficient mice or pharmacological inhibition of HSP70 both reduced graft infiltration with mo-DC after allogeneic transplantation in bone marrow (BM) plug and kidney transplantation models (8, 9). Additionally, it reduced kidney rejection severity. Our results indicate that HSP70 is a downstream mediator of innate allorecognition by monocytes with measurable impact on transplant rejection in mouse models. More specific pharmacological inhibition of HSP70 could have potential to be developed as a treatment option for chronic rejection.

## Results

### HSP70 is a key regulator downstream of monocyte allorecognition.

To gain insight into transcriptional changes of monocytes after innate allorecognition, we analyzed published scRNA-seq datasets from mice (GSE147596) (9) and human kidney allograft biopsies (E-MTAB-12051) (10) to identify upregulated genes after monocyte allorecognition. Datasets were reprocessed.

The scRNA-seq dataset GSE147596 consists of splenic monocytes sorted from B6-Rag2^−/−^ Il2rg^−/−^ mice 1 week after either allogeneic or syngeneic injection of 20 million irradiated BALB, C3H, or B6 splenocytes (9). Four unique neighborhoods were visualized in the UMAP (Figure 1A). Initially, we classified these neighborhoods based on the expression of Ly-6C encoded by *Ly6c2*, revealing Ly-6C^hi^, Ly-6C^int^, and Ly-6C^lo^ subsets (Figure 1B). Further profiling for antigen presentation–related gene expression showed that the Ly-6C^int^ subset is transitioning to mo-DC, as it differentially expressed the highest levels of MHCII (*H2-Ab1*) and CD74 (*Cd74*) as well as an intermediate level of CD11c (*Itgax*) (11, 12). One of the Ly-6C^lo^ subsets expressed the highest levels of CD36 (*Cd36*) and CD11c, supporting its transition to macrophages, given CD36’s role in phagocytosis.

After classification of monocyte subtypes, we performed differential gene expression analysis comparing Ly-6C^hi^ monocytes in Balb/c versus B6-injected groups. Differentially expressed genes (DEG) (|fold change > 1.5| and FDR < 0.01) were used to perform Qiagen’s Ingenuity Pathway Analysis (IPA) in order to pinpoint upstream regulators that are differentially expressed after allogeneic stimulation. Only 4 upstream regulators were shortlisted (Figure 1C). *Hspa1a* was one of the shortlisted upstream regulators, and its downstream targets included *Ptk2*, *Tnf*, *Xbp1*, and *Cdk1*. We validated that *Hspa1a* upregulation was not restricted to Ly-6C^hi^ monocytes recognizing allogeneic Balb/c splenocytes, but we also found that it was upregulated in Ly-6C^hi^ and Ly-6C^int^ transition to mo-DC subsets in Balb/c and C3H splenocyte–injected groups (Figure 1D). Notably, upregulation of *Hspa1a* was higher in the Balb/c as compared with the C3H group, consistent with our previous reports of the monocyte response to different allogeneic sources of injected splenocytes (8). This upregulation was absent in the Ly-6C^lo^ transition to macrophage subset, supporting its specific involvement in monocyte to mo-DC differentiation. To pinpoint which gene products are directly interacting with HSPA1A, we queried *Hspa1a*’s downstream targets and the other upstream regulators in STRING, an online database for protein-protein interaction. Only XBP1 and TNF directly interact with HSPA1A, as highlighted in Figure 1E. In addition, XBP1 is known to be required for DC development and survival (13). To note, *Xbp1* was upregulated in Ly-6C^hi^ monocytes after allostimulation with a fold change of 1.7; however, it did not reach significance (*P* = 0.15 and FDR step up = 0.47). Nevertheless, HSPA1A might be interacting with XBP1 during monocyte to DC differentiation. To confirm that the DEG in Ly-6C^hi^ monocytes after injection of irradiated Balb/c or C3H monocytes are specific to allostimulation, we performed bulk RNA-seq on BM-derived monocytes after nonspecific GM-CSF–driven monocyte to DC differentiation, comparing *Hspa1a* shRNA knockdown versus controls. Only 34 genes were both downregulated after shRNA knockdown and upregulated specifically after allostimulation by injection of irradiated Balb/c splenocytes (Supplemental Figure 1, A and B; supplemental material available online with this article; https://doi.org/10.1172/jci.insight.197348DS1). None of these genes are linked to monocyte differentiation to DCs, suggesting that the observed transcriptional changes in the Ly-6C^hi^ subset are specific to innate allorecognition.

To investigate if similar transcriptional changes can be observed in human transplant recipients, we analyzed a publicly available scRNA-seq dataset generated from kidney allograft biopsies (BioStudies accession no, E-MTAB-12051) of patients with transplant rejection (R, *n* = 4) and no rejection (NR, *n* = 12) (10). We classified the dataset’s major cell types using the Automated Garnett Cell Classifier (14). Only 1 unique neighborhood’s cells were labeled as myeloid (Supplemental Figure 2A). In addition, the myeloid classified neighborhood was the only *PTPRC*-expressing (CD45-expressing) neighborhood that also abundantly expressed *HSPA1A*. We selected this neighborhood and performed a dimensionality reduction and projection using t-SNE. This revealed the heterogeneity of the myeloid population as 7 distinct neighborhoods (Figure 2A). The cells were then classified by the expression of known unique markers for myeloid subsets (Supplemental Figure 2B). This allowed us to label the neighborhoods as cDC1, cDC2, mo-DC, M1 macrophages (MΦ), M2 MΦ, classical monocytes, and nonclassical monocytes (Figure 2A). Mo-DC and nonclassical monocytes were only detected in the R group, and M2 MΦ were only observed in the NR group (Figure 2B). This is consistent with an ongoing robust alloresponse in the R group and a dominant reparative response in the NR group. Supplemental Figure 2A shows that *HSPA1A* expression in immune cells was absent in B cells, minimal in T cell subsets and prominent in myeloid cells. We then profiled the myeloid subsets for differential expression of *HSPA1A* while comparing the 2 patient groups. Classical monocytes and cDC1, which are known to activate T cells, upregulated *HSPA1A* expression while cDC2, and M2 MΦ downregulated it during rejection (Figure 2C). Moreover, *HSPA1A* transcripts were present in mo-DC, a myeloid subset exclusively found in kidney biopsies in the rejection group, and in M2 MΦ, a myeloid subset exclusively detected in the nonrejection group. Upregulation cannot be assessed for either subset, owing to their absence in the comparator condition. We confirmed that this transcriptional upregulation is reflected at the protein level by intracellular HSP70 staining of graft-infiltrating monocytes (Supplemental Figure 3).

### Blocking the HSP70 pathway diminishes innate allorecognition.

To test the effect of blocking the HSP70 pathway on innate allorecognition, we used the allogeneic BM plug transplantation model, in which the intact BM cylinder from the femur of a donor mouse was transplanted under the kidney capsule of the recipient mouse (5, 8, 9). We used 2 different approaches. First, we blocked HSP70 by treating recipients with the HSP70 inhibitor deoxyspergualin (DSG) in recipients lacking all lymphoid cells (B6 Rag-gc–KO) to investigate the effect on innate allorecognition in the absence of adaptive immunity. DSG is a synthetic analogue of spergualin that engages the conserved C-terminal EEVD motif and the N-terminal nucleotide-binding domain of cytosolic HSP70 family members, inhibiting ATPase activity and downregulating *Hspa1a* expression (15–17). NOD BM plugs (Figure 3A) or Balb/c BM plugs (Figure 3B) were transplanted under the kidney capsule and procured on day 7 after transplantation. Flow cytometric analysis of the graft infiltrate demonstrated the presence of mo-DC in untreated recipients, both for the NOD and the Balb/c graft. Treatment of recipients with DSG, administered daily i.p., resulted in significant reduction of infiltration with mo-DC in the graft, indicating that blocking HSP70 with DSG impairs innate allorecognition by monocytes.

We then investigated if innate allorecognition is impaired in HSP70-deficient WT recipients. NOD BM plugs were transplanted under the kidney capsule of either WT or HSP70-KO recipients and graft infiltrate analyzed by flow cytometry. HSP70-KO recipients displayed significantly reduced infiltration of BM plugs with mo-DC compared with WT recipients (Figure 3C), indicating that HSP70 is a downstream regulator of innate allorecognition.

### Interfering with the HSP70 pathway improves renal transplant outcomes in mice.

To investigate if the HSP70 pathway is relevant in a more clinically relevant transplantation setting, we utilized an allogeneic kidney transplant model in mice and transplanted allogeneic Balb/c kidneys to B6 recipients. This model results in slow, chronic rejection with long-term survival and histopathological hallmarks of chronic rejection. Recipients were either WT or constitutive HSP70-deficient mice. An additional group received treatment with DSG i.p. for 60 days. Syngeneic kidney transplants served as controls. Since the kidney transplants were life-sustaining, survival was monitored. Grafts were procured at day 120 after transplantation, and histopathology and the renal immune infiltrate were assessed. Survival curves of all groups were not significantly different, although some allografts were lost in the WT and HSP70 groups due to urinary obstruction (Figure 4A). Histopathological Banff scoring of kidney graft rejection showed significantly lower Banff scores in the HSP70-KO and DSG-treated groups, compared with the untreated WT control group (Figure 4B). Photomicrographs of kidney grafts showed a visually higher immune infiltrate in the WT group compared with the HSP70-KO and DSG-treated groups (Figure 4C). Quantitation of fibrosis in kidney grafts showed no significant differences across groups, but quantitation of the immune infiltrate was significantly lower in the HSP70-KO and DSG-treated groups compared with the untreated WT group and similar to syngeneic grafts (Figure 4B). Although chronic rejection of kidney grafts in this model is not dependent on donor-specific antibodies, we measured DSA in all groups to determine if HSP70 deficiency or blockade confers any differences in alloantibody production. Figure 4D shows no differences in DSA production between all groups, indicating that there is no direct or indirect effect of blocking HSP70 on DSA production. To further characterize the differences in immune infiltrate in the absence of HSP70, we analyzed the phenotype of the immune infiltrate of the grafts by multicolor spectral flow cytometry at day 120 (Supplemental Figure 4). Infiltrating monocytes were not different between groups, but there were significantly reduced numbers of mono-DC in the HSP70-KO group and significantly less mature (CD80^+^MHCII^+^) mono-DC in both the HSP70-KO and DSG-treated group. Allograft infiltration with CD8 and CD4 T cells was significantly reduced in HSP70-KO recipients but did not reach significance in the DSG-treated group. Overall, these findings suggest that blocking HSP70 blocks maturation of mono-DC and causes a reduction in graft-infiltrating T cells.

To confirm that *Hspa1a* mRNA upregulation is reflected at the protein level and to localize HSP70 protein expression, we measured intracellular HSP70 by flow cytometry in lymphoreplete and HSP70-KO kidney transplant recipients at day 7 (Supplemental Figure 3). Graft-infiltrating monocytes from WT recipients expressed significantly higher intracellular HSP70 in kidney transplant and spleen, whereas HSP70 was absent in HSP70-KO recipients (Supplemental Figure 3B), confirming the HSP70-KO genotype. Within WT grafts HSP70 protein was expressed predominantly by myeloid subsets (Supplemental Figure 3C). Characterization of the graft immune infiltrate at day 7 showed significantly reduced T cell (*P* = 0.0004) and CD8 T cell (*P* = 0.0053) numbers in HSP70-KO compared with WT recipients (Supplemental Figure 3D), consistent with the reduced T cell infiltration observed in the chronic kidney transplant model (Figure 4E). To assess whether HSP70 deficiency alters allospecific T cell priming, we enumerated allospecific CD8 T cells using a pooled H-2K(d) tetramer panel. Allospecific tetramer^+^ CD8 T cell numbers did not differ between WT and HSP70-KO recipients in either the graft (*P* = 0.1000) or spleen (*P* = 0.2000) at day 7 (Supplemental Figure 3E), indicating that initial allospecific CD8 priming is intact in the absence of HSP70 and that the reduced intragraft T cell infiltrate reflects a graft-local rather than a systemic priming defect. Overall immune subset composition in the spleen of naive WT and HSP70-KO mice was similar (Supplemental Figure 3F), whereas NK and monocyte numbers were significantly reduced, and macrophage numbers increased, in HSP70-KO mice 7 days after allosensitization, consistent with a predominantly myeloid-restricted effect (Supplemental Figure 3F).

To further test the role of the HSP70 pathway in innate allorecognition, we generated BM-derived immature DC in vitro. Immature BMDC from either B6 WT or B6 HSP70-KO mice were cocultured with congenic B6 syngeneic or Balb/c allogeneic splenocytes for 5 days. Flow cytometric analysis for mature (CD80^+^MHCII^+^) BMDC showed similar maturation of BMDC in both WT and HSP70-KO groups after LPS stimulation (Figure 4F). B6 BMDC matured after allogeneic stimulation to a similar extent than LPS stimulated WT BMDC. Maturation of HSP70-KO BMDC was significantly impaired after allogeneic stimulation (*P* ≤ 0.0001) compared with WT BMDC (Figure 4G). These data show not only that the HSP70 pathway plays a role in maturation of monocyte-derived DC after allorecognition but also demonstrates that pathways causing maturation of BMDC after LPS stimulation are not dependent on HSP70.

## Discussion

HSP70 has been reported to trigger activation of DC, thus perpetuating a strong inflammatory immune response (18). In addition, higher levels of the inducible form of HSP70 have been detected in allogeneic transplanted rat hearts as compared with isografts (19). Inducible HSP70-mediated signaling in human monocytes leads to enhanced activation and production of proinflammatory cytokines (20, 21). Moreover, in vitro recombinant HSP70 induces DC maturation (22).

DSG binds to HSP70’s EEVD domain, inhibits HSP70 ATPase activity, and downregulates *Hspa1a* expression (15–17). It has also been shown to specifically inhibit myeloid cell activation, proliferation, their capacity to activate T cells, and lack of a direct effect on T cells in both in vitro immunization experiments and in vivo transplant models (23–26). In nonhuman primates, short-term treatment with DSG and cyclosporine led to significant increases in survival of heart or kidney transplant recipients (27). In human trials, short-term treatment of DSG initiated early after kidney transplantation with T cell depletion using alemtuzumab did not induce tolerance after treatment cessation (28). However, in kidney transplant patients suffering from steroid-resistant or late rejection, short-term DSG treatment is as effective as anti–T cell monoclonal antibody (OKT3) or steroid treatment, respectively, in improving kidney function (29, 30).

Our analysis of the scRNA-seq dataset from kidney allograft biopsies elucidated considerable heterogeneity within the myeloid cell populations. Utilizing t-SNE, we identified 7 distinct myeloid neighborhoods, with mo-DC and nonclassical monocytes present exclusively in the R group, and M2 macrophages (MΦ) identified solely in the NR group. This differentiation underscores the divergent immunological landscapes between the 2 patient groups. HSP70 was highly expressed in myeloid cells within the kidney graft, whereas other immune cell subsets expressed little or no HSP70. Some kidney parenchymal cells also upregulated HSP70 expression. These findings highlight that, within immune cells, myeloid cells are the prime target of HSP70 blockade. *HSPA1A* transcripts were also detected in cDC2 and M1 macrophages, subsets present in both patient groups; these expression data reflect intracellular transcripts, and the functional significance of *HSPA1A* in these subsets in human kidney transplantation remains to be defined.

Further experiments showed that pharmacological inhibition of the HSP70 pathway, achieved through administration of the inhibitor DSG, resulted in a notable decrease of mo-DC infiltration within BM plug grafts, corroborated by findings in HSP70-deficient recipients. These observations indicate HSP70’s pivotal role downstream of innate allorecognition by monocytes. Furthermore, both DSG treatment and HSP70 deficiency were associated with improved renal transplant outcomes in mice, as evidenced by reduced Banff scores, lower immune cell infiltrate, and diminished presence of mature mono-DC and T cells within grafts. We further show that targeting HSP70 in this model does not impair maturation of DCs in response to LPS and should leave antimicrobial immune responses intact.

These findings align with existing literature that delineates HSP70’s involvement in DC activation and the maintenance of inflammatory responses. Elevated levels of inducible HSP70 in allografts, alongside its role in monocyte activation and cytokine production, have been well documented (18–22). The specific inhibitory effects of DSG on HSP70 ATPase activity, and its consequent effect on myeloid cell activation, are consistent with prior studies (15–17). An encouraging finding of our study is the effectiveness of DSG treatment on long-term outcomes of allografts despite restricting treatment to the first 8 weeks after transplantation and limiting administration to 3 times per week during later treatment. Since DSG has a short half-life, it has traditionally been administered daily in patients who have undergone renal transplant. This might also explain why some of the significant differences between WT and HSP70-KO groups were not detectable in the DSG group (treated for the first 8 weeks only), although similar trends were observed.

The findings of this study are also consistent with our previous work demonstrating that monocytes recognize allogeneic nonself and contribute to allograft rejection. The key receptors we have identified so far are the SIRPa-CD47 and PIR-A–MHC-I receptor pairs, which are essential for primary and memory monocyte responses to allogeneic nonself and promote differentiation to mo-DC (8, 9). Previous work from our group has demonstrated that migration of effector and memory T cells to the graft is an antigen-dependent process that is governed by mo-DC in the graft (5, 31, 32). These mo-DC not only present antigen to facilitate infiltration of the graft with T cells but also maintain local immune responses by presenting antigen and cytokines necessary for survival of tissue-resident memory T cells in the graft (4). This central role of monocyte-derived DC in the allograft explains why a defect in mo-DC maturation through targeting HSP70 results in a lower T cell infiltrate and better graft outcomes in our mouse kidney transplant model.

### Limitations of our study

While our protein-level data demonstrate that HSP70 is present and upregulated intracellularly within graft-infiltrating monocytes (Supplemental Figure 3, B and C), they do not address whether HSP70 is also secreted. The intact LPS-induced maturation of HSP70-KO BMDC (Figure 4F) indicates that TLR4 signaling is preserved and that the allorecognition-associated maturation defect (Figure 4G) operates independently of the extracellular HSP70/TLR2/4 pathway described for soluble HSP70. A limitation of this study is the use of constitutive HSP70-deficient mice, which lack HSP70 in all lineages. However, naive HSP70-KO mice showed normal immune subset composition. After allo-immunization, splenic T and B compartments were equivalent between genotypes, and allospecific CD8 priming was intact (Supplemental Figure 3E). Together with the myeloid-predominant expression of HSP70 within the graft (Supplemental Figure 3C), intact monocyte recruitment with impaired mo-DC differentiation (Figure 4E), and the cell-intrinsic maturation defect in vitro (Figure 4G), these observations localize the functionally relevant effect to graft myeloid cells. Generation of a myeloid-specific conditional KO would be required to establish this definitively.

Our study advances the understanding of the critical function of the HSP70 pathway in innate allorecognition and underscores its potential as a therapeutic target of more specific inhibitors to enhance transplant outcomes.

## Methods

### Sex as a biological variable.

Both sexes of mice were used, but males were preferred for the transplantation procedure due to size and anatomy. Previous studies have not identified sex differences in allograft rejection beyond the known H-Y minor histocompatibility Ag in the absence of an MHC mismatch. The findings obtained in this study are expected to be relevant to both sexes.

### Study design.

Three biological replicates (3 individual transplant recipients) per group were included in each experiment. Experiments were repeated at least once, resulting in a total of 6–10 biological replicates. Sample sizes were based on prior observations that 6 biological replicates were sufficient to discern statistically significant differences between groups, with observed effect sizes > 0.5. Prospective exclusion criteria were transplant recipient death within the first 7 days after transplantation (technical failure) and urinary obstruction (censored data points). All other data points were included, and no outliers were excluded. All end points were prospectively selected. It was not possible to blind the study because of the need to identify donors and recipients. Histopathological scoring was blinded.

### Animals.

Balb/cJ (Thy 1.2, CD45.2), B6.CD45.2 (C57BL/6J; Thy1.2, CD45.2), B6.CD45.1 (B6.SJL-*Ptprc^a^Pepc^b^*/BoyJ, Thy1.2, CD45.1, NOD (NOD/ShiLtJ), Balb/c CD45.1 (CByJ.SJL(B6)-Ptprca/J), and Balb/c Rag-gc-KO (C;129S4-Rag2tm1.1Flv Il2rgtm1.1Flv/J) were from Jackson Laboratory (Jax). B6 Rag-gc-KO (#4111, C57BL/6NTac.Cg-Rag2tm1Fwa Il2rgtm1Wjl) were from Taconic. B6.CD45.1 (B6.SJL-*Ptprc^a^Pepc^b^*/BoyCrl) were from Charles River Laboratories. B6 *Hspa1a/Hspa1b*^-/-^ (Hsp70.1/Hsp70.3 double KO; referred to here as HSP70-KO) mice were obtained from The Methodist Hospital Research Institute under an MTA with The Washington University (33).

### Kidney transplantation and pharmacological treatment.

Mouse kidney transplants were performed as previously described (34). Recipient native kidneys were removed during the transplantation procedure. Allograft rejection was monitored by visual observation of recipients for signs of uremia (lethargy, decreased mobility, and ruffled hair) or death. Some groups were treated with DSG, 5 mg/kg i.p. daily for the first 2 weeks, followed by 1 mg/kg 3x/week for the remaining 6 weeks.

### Histologic analysis.

Kidney allograft tissue was fixed in formalin, paraffin-embedded, sectioned, and stained with H&E, Masson’s trichrome (MT), and periodic acid–Schiff (PAS) stain (Magee-Women’s Research Institute Histology and Microimaging Core, University of Pittsburgh). All slides were scanned on a Zeiss Axioscan.Z1 with a 20× objective and analyzed in QuPath (35). A pixel classifier was trained in QuPath to quantitate immune infiltration and fibrosis per kidney section at time of rejection, using H&E- and trichrome-stained sections, respectively. Histological sections of allografts were scored according to Banff classification. Banff grades were assigned numerical values: normal = 0, borderline = 0.5, IA = 1.0, IB = 1.5, IIA = 2.0, IIB = 2.5 and III = 3.0.

### BM plug transplantation and pharmacological treatment.

BM plug transplantation was performed under the kidney capsule after isolating intact BM plugs from donor femurs (5). Recipient mice were anesthetized, and the kidney was exposed via a small flank incision. A small incision was made in the kidney capsule, a pocket was created with blunt forceps, and a 4 mm BM plug fragment was placed in the subcapsular pocket with vascular forceps. Some groups were treated with DSG, 5 mg/kg i.p. daily. Depleting antibodies were procured from BioXcell: anti-CD4 (clone GK1.5, BE0003-1), anti-CD8 (clone 2.43, BE0061), anti-CD20 (clone MB20-11, BE0356), anti-NK1.1 (clone PK136, BE0036). Animals were treated with a single dose of 250 μg i.p. in experiments as indicated.

### Leukocyte isolation and flow cytometry.

Graft tissue was processed as follows: BM plugs were removed from the kidney capsule; kidney grafts were removed from the abdominal cavity. Graft tissue was homogenized using a gentleMACS tissue processor (Miltenyi), and digested at 37°C for 45 minutes in RPMI plus 10% fetal calf serum containing collagenase IV (350 U/mL; Sigma-Aldrich) and deoxyribonuclease I (20 ng/mL; Sigma-Aldrich). Leukocytes were isolated by gradient centrifugation using Lympholyte-M (Cedarlane Laboratories). Total recovered cells were counted using an automated cell counter (Beckman Coulter) before staining with antibodies. Fluorochrome- or biotin-tagged antibodies were purchased from BD Pharmingen, eBioscience, BioLegend, or R&D Systems: CD90.2 (30-H12), CD8 (53-6.7), CD4 (GK1.5), CD45.1 (A20), CD45.2 (clone 104), CD45R/B220 (RA3-6B2), CD49b (DX5), NK1.1 (PK136), F4/80 (BM8), CD11b (M1/70), CD11c (N418), Ly6G (1A8), CD19 (1D3), MHC II (M5/114.15.2), and CD80 (16-10A1). Fixable Viability Dye Blue was purchased from Invitrogen/Fisher (L34961). Flow acquisition was performed on a 5-laser Cytek Aurora multispectral flow cytometer (Cytek), and data were analyzed using FlowJo software (Tree Star Corp.). Recipient and donor cells were distinguished using congenic markers (CD45.1/2),

### Intracellular HSP70 staining.

For intracellular HSP70 detection, leukocytes isolated from kidney grafts and spleens on day 7 after transplant were stained for viability and surface markers, then fixed and permeabilized using the eBioscience Foxp3/Transcription Factor Staining Buffer Set according to the manufacturer’s instructions. Cells were stained intracellularly with anti-HSP70 antibody (clone C92F3A-5, FITC, abcam, ab61907) and an IgG1 isotype control (Invitrogen, 11-4714-81, clone P3.6.2.8.1, FITC). The frequency of HSP70^+^ cells was determined within each gated immune subset.

### Allospecific CD8 T cell detection by H-2K(d) tetramer staining.

Allospecific CD8 T cells in graft and spleen at day 7 were identified using a pooled panel of 14 PE-conjugated H-2K(d) tetramers loaded with allopeptides (SYFPEITHI, HFLPMLQTV, KYIHSANVL, SYHPALNAI, NYFPSKQDI, GYFEVTHDI, AYAPSGNFV, KYSEVFEAI, GYLPLAHVL, YYLNDLERI, NYISGIQTI, SYLPPGTSL, NYLPAINGI, and VYSNTIQSI; panel developed by Alexandra Sharland, The University of Sydney) (36), provided by the NIH Tetramer Core Facility, Emory University. Single-cell suspensions were incubated with the pooled tetramer panel and crosslinked with a purified anti-PE antibody prior to surface antibody staining, and tetramer-positive events were enumerated within gated CD8 T cells.

### Reprocessing and bioinformatic analyses of published scRNA-seq data.

We reprocessed 2 publicly available scRNA-seq datasets using Cell Ranger (v8.0) according to developer’s instructions. The first dataset was generated in-house from sorted splenic monocytes after allogeneic stimulation or syngeneic injection of irradiated splenocytes (Gene Expression Omnibus accession number GSE147596) in B6 Rag-gc-KO mice, which lack T, B, and NK cells. The second dataset was generated by Martin Naesens’ group from kidney allograft biopsies of rejecting and nonrejecting patients (BioStudies, accession no. E-MTAB-12051). The resultant filtered matrix files were uploaded to Partek Flow (v12.1.0). Both datasets were further quality controlled for dead or apoptotic cells (%mitochondrial genes expressed < 20%) and doublets (mRNA counts < 15,000 and detected features < 4,000) then normalized by log_2_(counts per million + 1). For the mouse dataset, Uniform Manifold Approximation and Projection (UMAP) was performed based on the top 10 principal components; then, neutrophils and eosinophils were excluded based on cellular neighborhood’s expression of *Ly6g* and *Siglecf*. Initial dimensionality reduction was performed using Principal Component Analysis of monocytes, performed on the top 2,000 genes by variance of gene expression. UMAP of the top 5 principal components projected monocyte neighborhoods that were characterized based on the expression of myeloid cell type unique genes (Figure 1, A and B). The choice of number of principal components to use for UMAP was dependent on elbow point of Eigenvalue scree plot. For the patient dataset, after normalization, we removed unwanted sources of variation between samples using Seurat3 integration. We then projected the cells in 2D space using t-SNE on the top 10 principal components and utilized Garnett Classifier for automated cell type annotation (Supplemental Figure 2A). The neighborhood of cells classified as myeloid cells was then filtered in for reprojection using t-SNE on top 8 PCs. The resultant neighborhoods were classified based on expression of markers that allow for the distinction between cDC1, cDC2, Classical and Nonclassical monocytes, M1 and M2 macrophages, and mo-DC (Supplemental Figure 2B). For both datasets, differential gene expression analysis was done using GSA (Gene Specific Analysis, Partek) (|fold change| > 1.5 and FDR < 0.1). Upstream regulator identification and Protein-Protein Interaction Networks analysis was performed using IPA and STRING, respectively.

### shRNA HSP70 knockdown and Bulk RNA-seq from BMDC.

BMDCs were subjected to total RNA extraction utilizing the TRIzol reagent (Invitrogen), in strict adherence to the manufacturer’s guidelines. The purity and concentration of the RNA were evaluated with a NanoDrop ND-1000 spectrophotometer (Thermo Fisher Scientific), and its integrity was confirmed via agarose gel electrophoresis. For bulk RNA-seq, 1 μg of total RNA per sample was employed in library preparation. Prior to this, DNase I treatment was applied to eliminate any residual DNA. The RNA was then fragmented, and cDNA synthesis was performed using random hexamer priming. Subsequent steps included end-repair, A-tailing, and adapter ligation to prepare the cDNA libraries. These libraries underwent PCR amplification, followed by quality and quantity validation using the Agilent 2100 Bioanalyzer (Agilent Technologies) and the Qubit 2.0 Fluorometer (Life Technologies). High-throughput sequencing was executed on NovaSeq 6000. Briefly, raw sequencing reads were first assessed for quality using FastQC, and adapter sequences and low-quality bases were removed using Trim Galore. The resulting clean reads were aligned to the mouse reference genome (GRCm39/mm39) using STAR with default parameters, and gene-level read counts were quantified with featureCounts against the corresponding Ensembl/GENCODE gene annotation. Genes with consistently low expression across all samples were filtered out prior to downstream analysis. Raw read counts were normalized using the median-of-ratios method implemented in DESeq2. To assess the transcriptional impact of HSP70 (*Hspa1a/Hspa1b*) knockdown, differential expression analysis was performed between shHSP70 and shControl BMDCs using DESeq2. *P* values were adjusted for multiple comparisons using the Benjamini-Hochberg method, and genes with an adjusted *P* < 0.05 and an |Log_2_ fold change| > 1 were considered significantly differentially expressed.

### Donor-reactive antibody detection.

Donor-specific antibodies were detected by incubating recipient serum with donor splenocytes and detecting bound antibodies with anti-mouse IgG-FITC antibody (Life Technologies, 11-4011-85). Briefly, donor splenocytes were incubated with 20% FBS for 20 minutes at room temperature to block nonspecific binding. Recipient serum (25 μL) was added to 0.5 × 10^6^ donor splenocytes and incubated on ice for 1 hour. Cells were washed and surface staining for CD3-PE (eBioscience, clone 145-2C11), B220-eF450 (eBioscience, clone RA3-6B2), and anti-IgG performed. Samples were acquired on a BD Fortessa or Cytek Aurora spectral cytometer. Alloantibody binding was assessed on T cells and MFI reported.

### In vitro culture and stimulation of BMDC.

For generation of BMDC, BM was collected from femurs and tibias of either B6 WT or B6 HSP70-KO mice, and after RBC lysis with ACK lysis buffer, BM cells were cultured for 3 days in RPMI supplemented with 10% FBS and 20 ng/mL GM-CSF. On day 3, BMDC were cultured with either syngeneic or allogeneic (Balb/c) splenocytes in a 1:5 ratio. Control BMDC were cultured with media supplemented with LPS and IL-4.

### Statistics.

Statistical analysis was performed using Prism v.9 (GraphPad). Comparisons between 2 groups were analyzed either by unpaired 2-tailed Student’s t-test (Supplementary Fig. 3B, E) or, where data were not assumed to follow a normal distribution, by the 2-tailed nonparametric Mann–Whitney *U* test (Fig. 3A–C). Comparisons among three or more groups with a single independent variable were analyzed by 1-way ANOVA followed by Tukey’s multiple comparisons test (Fig. 4B, D, E). Datasets with 2 independent variables were analyzed by 2-way ANOVA followed by Tukey’s multiple comparisons test (Fig. 4F, G and Supplementary Fig. 3C, D, F). Renal allograft survival was compared using the log-rank (Mantel–Cox) test (Fig. 4A). All *P* values, regardless of statistical significance, are reported, and a *P* value less than 0.05 was considered significant. Group sizes (*n*, number of biologically independent animals or samples) and the number of independent experiments performed (*N*) are stated in each figure legend.

### Study approval.

All animal experiments were performed with approval and under supervision of the IACUC of the University of Pittsburgh, protocol no. 24014316 and 23103669, Animal Welfare Assurance, no. D16-00118 (A3187-01).

### Data availability.

The scRNA-seq datasets reanalyzed in this study are publicly available: mouse splenic monocytes under Gene Expression Omnibus accession GSE147596, and human kidney allograft biopsies under BioStudies accession E-MTAB-12051. The shRNA HSP70 knockdown bulk RNA-seq dataset is available under Gene Expression Omnibus accession GSE343161. All values underlying the graphs and reported means in the manuscript and supplement are provided in the Supporting Data Values file. Additional data are available from the corresponding authors upon reasonable request.

## Author contributions

MHO, KIAD, and FS conceived studies and designed experiments. NF performed experiments and data analysis. ZG performed transplant procedures and participated in experiments and scRNA-seq analysis. BD performed experiments and data analysis. CL performed transplant procedures. HD performed all BM plug transplantation experiments. AF performed mouse scRNA-seq experiments and analysis. SMS performed reanalysis of published RNA-seq datasets. MHO and KIAD analyzed, interpreted and reviewed all data. LH and ALW performed mouse colony management and sample processing, including flow cytometry and genetic typing of mice. MAM conducted the histological quantitation and PSR performed histopathological Banff scoring. MHO, KIAD, and FS wrote the manuscript. All authors contributed to reviewing and editing the manuscript. The order of co–first authors was determined based on who initiated the project.

## Conflict of interest

The authors have declared that no conflict of interest exists.

## Funding support

This work is the result of NIH funding, in whole or in part, and is subject to the NIH Public Access Policy. Through acceptance of this federal funding, the NIH has been given a right to make the work publicly available in PubMed Central.

- NIH grant AI181954 to MHO.
- 2020 Stuart K. Patrick Grant for Transplant Innovation and the 2023 American Society of Transplantation Career Transition Grant (grant no. 998676) to KIAD.
- ASN George B. Rathmann Research Fellowship program to ZG.
- American Society of Transplantation Sanofi Basic Science Fellowship Research Grant to NF.

## Supplementary Material

Supplemental data

Supporting data values

## Figures and Tables

**Figure 1:**
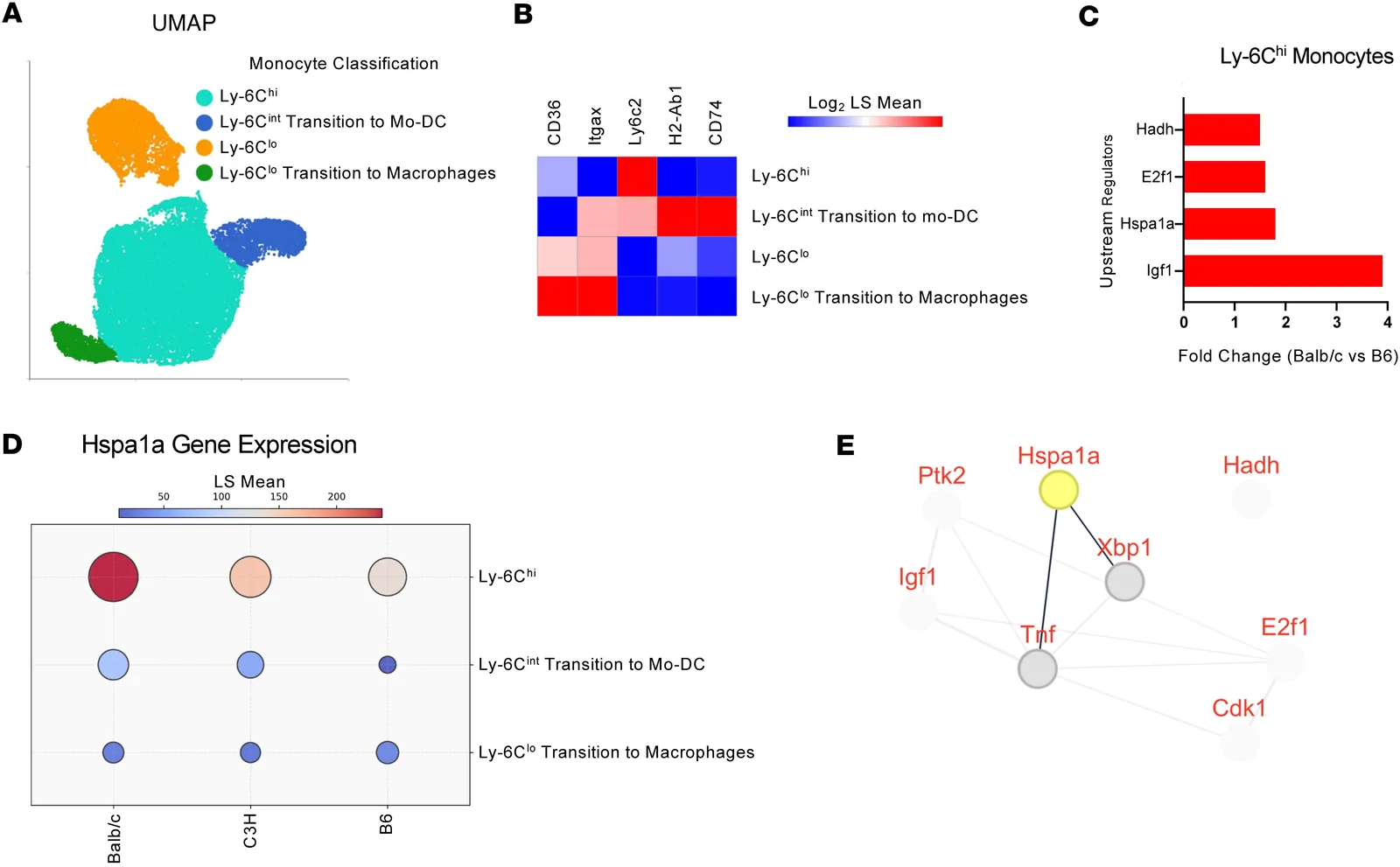
Inducible HSP70 is upregulated downstream of innate allorecognition. Analysis of our in-house published scRNA-seq dataset (GSE147596) of murine splenic monocytes. B6 Rag-gc-KO mice were immunized with allogeneic (Balb/c, C3H) or injected with syngeneic (B6) splenocytes and monocytes analyzed 1 week later as published (9). (**A**) UMAP of monocytes annotated by Ly-6C expression and antigen presentation gene signature. (**B**) Heatmap displaying expression of differentially expressed genes used to annotate monocytes. (**C**) Ingenuity analysis of upstream regulators differentially expressed after allostimulation. (**D**) Bubble plot displaying expression of *Hspa1a* by color scale and bubble size in monocyte subsets across groups. (**E**) STRING analysis of downstream targets of HSPA1A.

**Figure 2:**
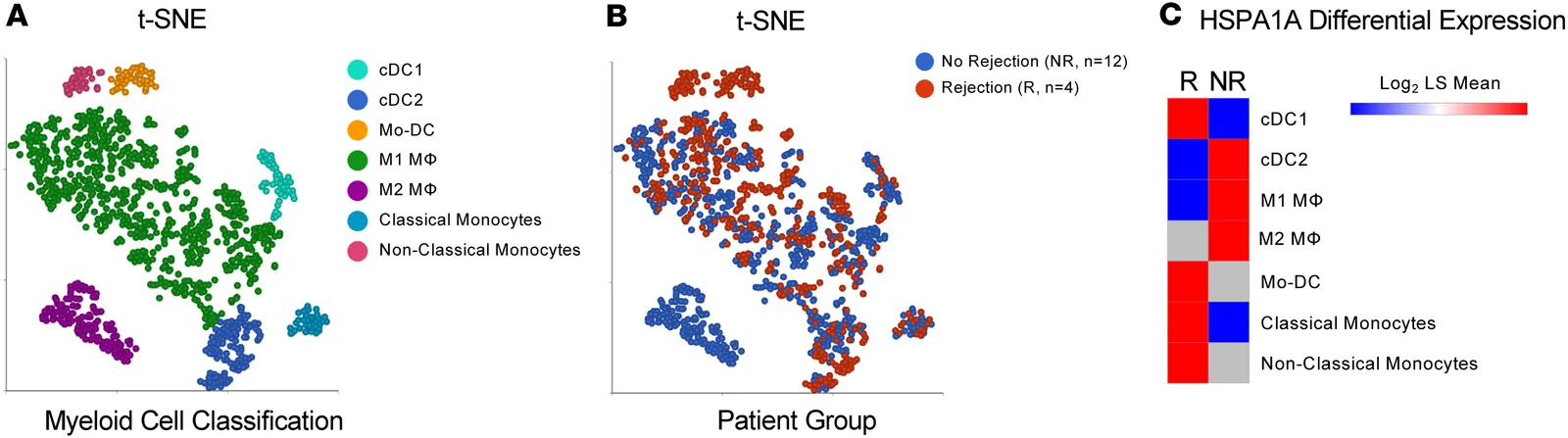
*HSPA1A* expression in myeloid cells in rejecting human kidney allografts. Analysis of myeloid cells infiltrating kidney transplant biopsies from published scRNA-seq dataset (E-MTAB-12051). (**A**) t-SNE of the myeloid cells with visualized classification from Supplemental Figure 2. (**B**) t-SNE of the myeloid cells highlighting their source from rejection or no rejection groups. (**C**) Heatmap visualizing expression of *HSPA1A* in the distinct myeloid subsets comparing rejection versus nonrejection groups. Color reflects *HSPA1A* expression: blue, low; red, high. For subsets present in both patient groups (cDC1, cDC2, classical monocytes, M1 macrophages), color represents relative expression between rejection and nonrejection. mo-DC (rejection only), M2 macrophages (nonrejection only) and nonclassical monocytes (rejection only) are present in a single group and have no comparator. For these, color reflects the level of transcript detected in that group and up- or downregulation cannot be inferred. Gray indicates subset absent from that group.

**Figure 3:**
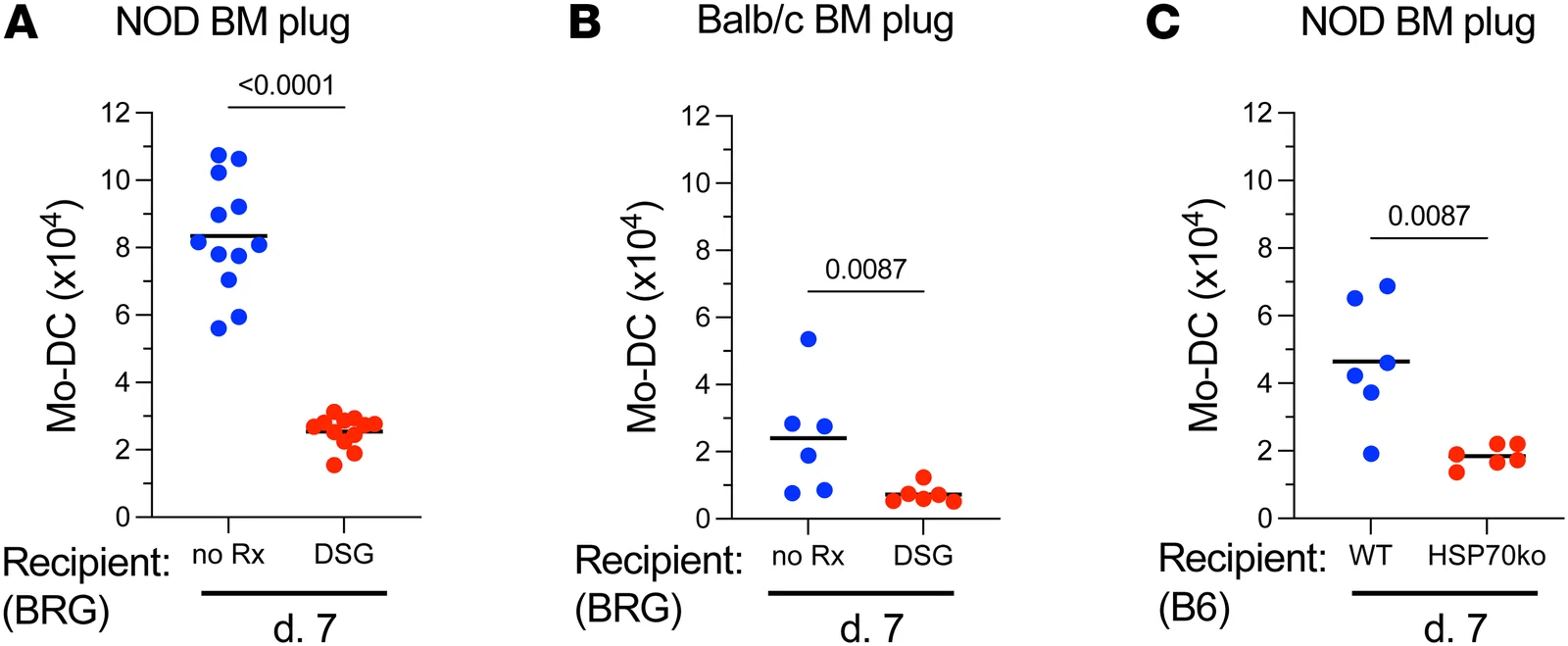
Blocking the HSP70 pathway reduces innate allorecognition. Bone marrow plugs from NOD or Balb/c donors were transplanted under the kidney capsule of recipient mice as indicated. BM plugs were removed and analyzed by flow cytometry on day 7 after transplantation. Mo-DC (lin^–^CD11b^+^F4/80^–^CD11c^+^) were enumerated. (**A** and **B**) NOD (*P* < 0.0001) and Balb/c BM plugs (*P* = 0.0087) transplanted to B6 Rag-gc-KO or B6 Rag-gc-KO recipients treated with deoxyspergualin (*n* = 6–12/group, *n* = 2 experiments). (**C**) Mo-DC infiltration of allogeneic NOD BM plugs in B6 WT and HSP70-KO recipients treated with anti-CD4, -CD8, -CD20 and -NK1.1 (*P* = 0.0087) (*P* < 0.05 considered significant, Mann-Whitney *U* test) (*n* = 6/group, *N* = 2 experiments).

**Figure 4:**
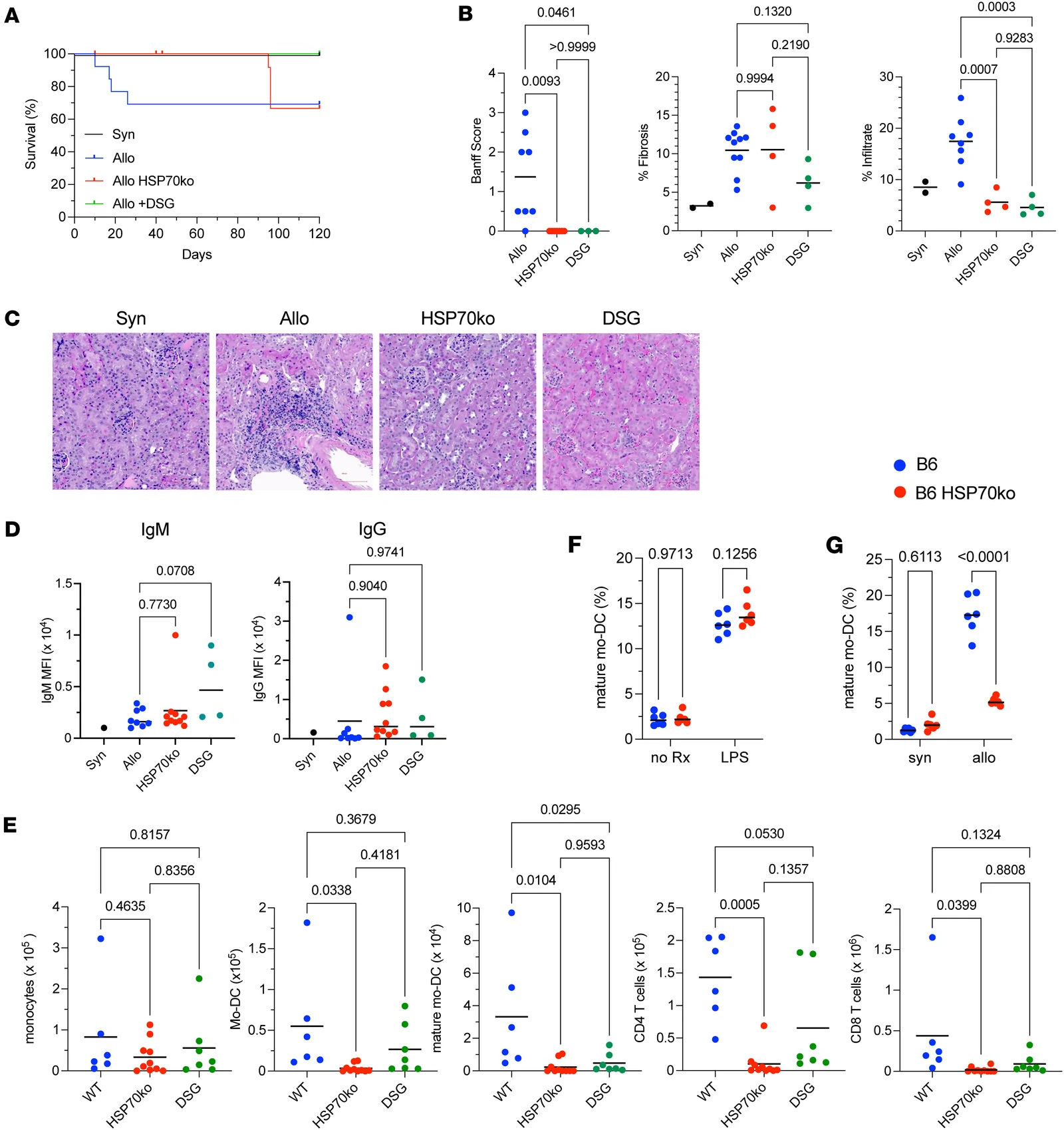
Interfering with the HSP70 pathway improves renal transplant outcomes. Balb/c kidneys were transplanted to B6 recipients as indicated. Syngeneic B6 KTx shown as controls (*n* = 2). Grafts were procured at day 120 after transplant (*n* = 4–8, *N* = 2–3 experiments). (**A**) Graft survival (log-rank test). (**B**) Quantitation of Banff score, fibrosis, and immune infiltrate of grafts is shown. (*P* < 0.05 considered significant, 1-way ANOVA). (**C**) Photomicrograph of H&E histology of indicated representative kidney graft. Scale bar: 100 μm. (**D**) MFI of serum donor-specific antibody measurements for IgM and IgG (*n* = 4–10, syn shown as baseline reference) (*P* < 0.05 was considered significant, 1-way ANOVA with Tukey’s multiple comparison test). (**E**) Quantitation of immune cell infiltrate in allografts (*P* < 0.05 was considered significant, 1-way ANOVA with Tukey’s multiple comparison test). (**F** and **G**) In vitro BMDC maturation assay. Maturation of B6 WT and HSP70-KO BMDC after LPS stimulation (**F**), and after coculture with syngeneic or allogeneic splenocytes (**G**) (*P* < 0.05 considered significant, 2-way ANOVA) (*n* = 6, *N* = 1 experiment).

## Version 1 10/08/2026

Electronic publication
