# Supplementary material for: Innate allorecognition induces HSP70 in monocytes contributing to renal allograft rejection
Source: JCI Insight. 2026 Oct 8;11(19):e197348. doi: 10.1172/jci.insight.197348 (PMC13726426; doi:10.1172/jci.insight.197348)
Supplement: Supplementary file 1 [file jciinsight-11-197348-s153.pdf]

A

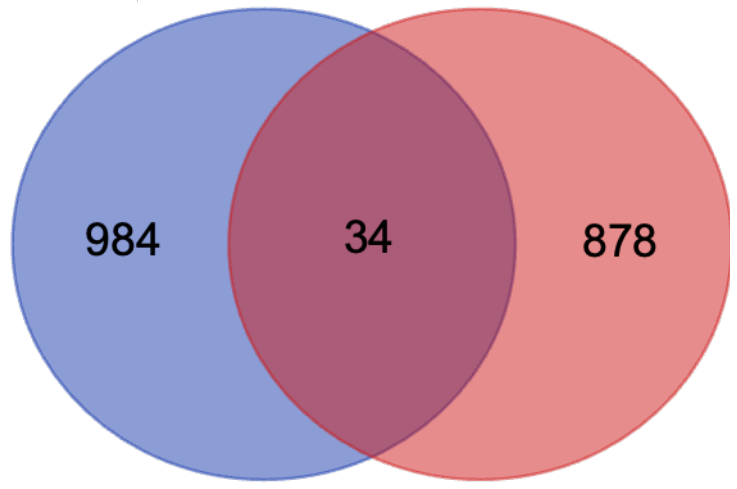


B

List of the common 34 genes from Venn Diagram in (A)

| | |
|---|---|
| Ppfibp1 | Gins2 |
| Hcar2 | Lilrb4a |
| Spef1 | 5830432E09Rik |
| Ccl5 | Gm21399 |
| Zfp646 | Hmga1b |
| Myo9b | Pfas |
| Homer3 | Slfn9 |
| Cd81 | Angptl2 |
| Ptges | Mms22l |
| Edil3 | Hmox1 |
| Vat1 | Stxbp1 |
| Orc1 | Eaf1 |
| Actn1 | Pidd1 |
| Arg1 | Jun |
| Tk1 | Clcf1 |
| Srxn1 | Mapk11 |
| Fancm | Cav2 |

Supplemental Figure 1: **HSP70 regulation of innate allorecognition is unrelated to GM-CSF driven monocyte to DC differentiation.**
(A) Venn Diagram of genes upregulated in Ly-6Chi splenic monocytes 7 days after allostimulation with injection of 20m irradiated Balb/c splenocytes and downregulated in shRNA HSP70 knockdown monocytes during GM-CSF driven monocyte to DC differentiation.
(B) List of the 34 common genes in the Venn diagram.

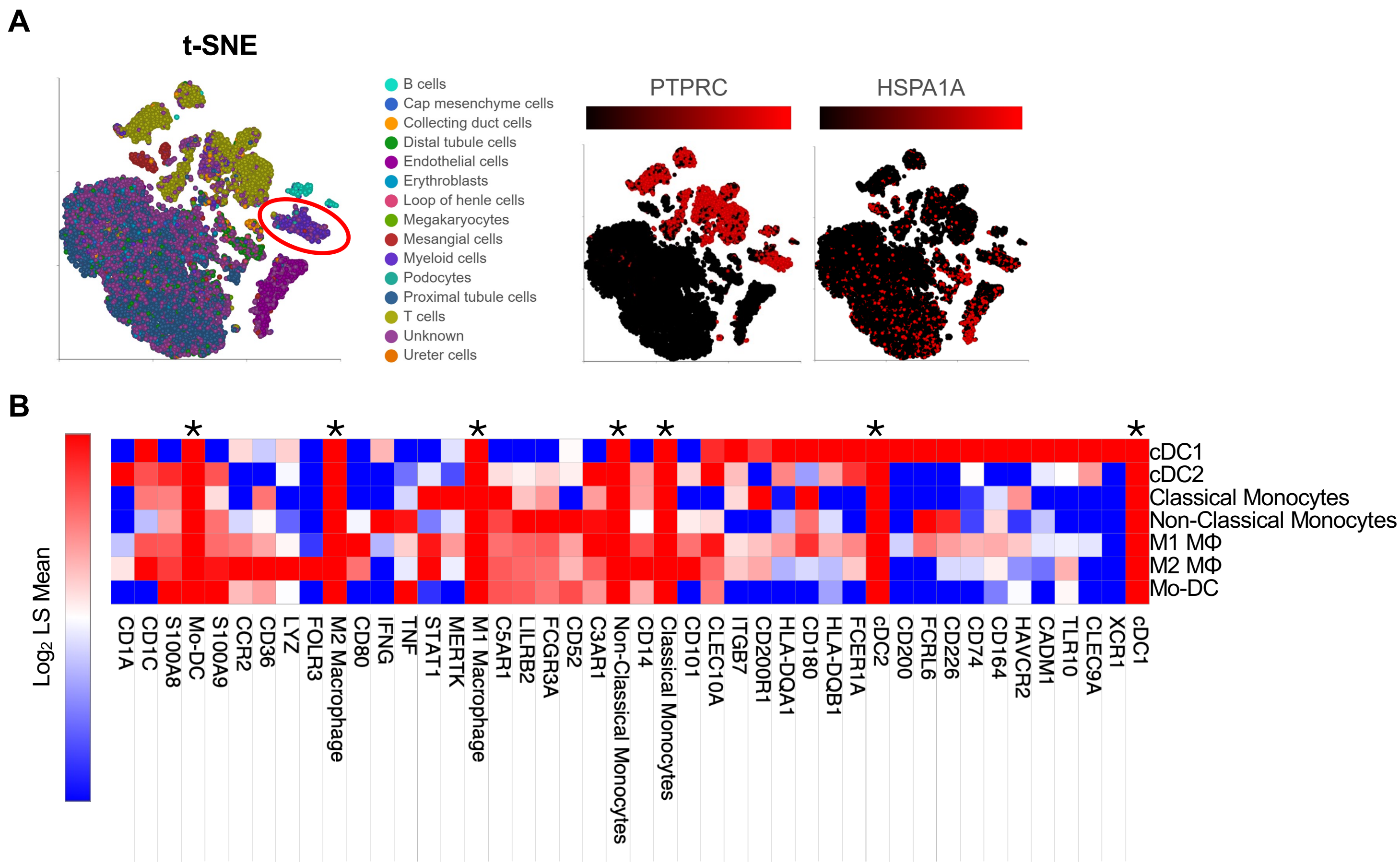


Supplemental Figure 2: **Classification of single cells in scRNAseq dataset E-MTAB-12051 from kidney transplant biopsies.**
(A) t-SNE of immune and non-immune cells from the dataset with classification from automated Garnett cell classifier highlighted, left panel, expression of PTPRC and HSPA1A visualized on the t-SNE, right panels. (B) Heatmap of expression of marker genes used to classify the myeloid neighborhoods of Figure 2.

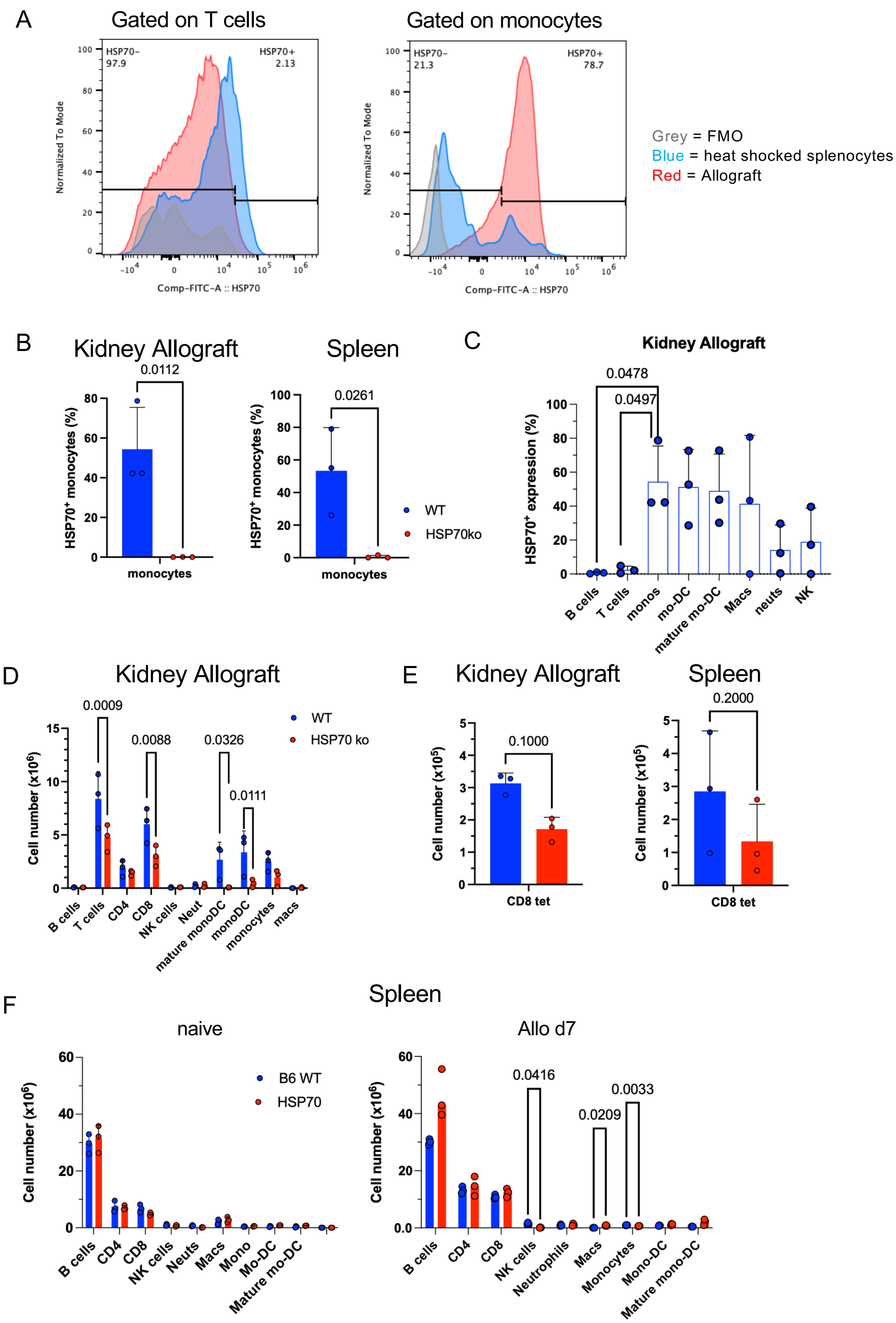


Supplemental Figure 3: **Immune subsets in WT and HSP70-ko mice.**
(A) Representative flow plots depicting intracellular HSP70 staining in WT cells. Gating according to Suppl. Fig. 4. Shown are fluorescence-minus-one control (grey), heat-shocked positive control (blue) and allograft sample. (B) Intracellular HSP70 expression in monocytes in KTx and spleen on d7. (C) Frequency of intracellular HSP70+ cells in allograft infiltrating immune subsets in WT mice on d7 post Tx. (D) Immune subsets in KTx on d7 after transplantation. (E) Number of allospecific (H-2K(d) tetramer panel positive) CD8 T cells in allograft and spleen on d7. (F) Immune subset characterization in the spleen comparing WT and HSP70-ko mice, left panel naïve mice, right panel Balb/c immunized mice on day 7. ($p<0.05$ considered significant, unpaired t-test (B, E) and two-way ANOVA with Tukey's multiple comparisons test (C, D, F))(n=3, N=2 experiments).

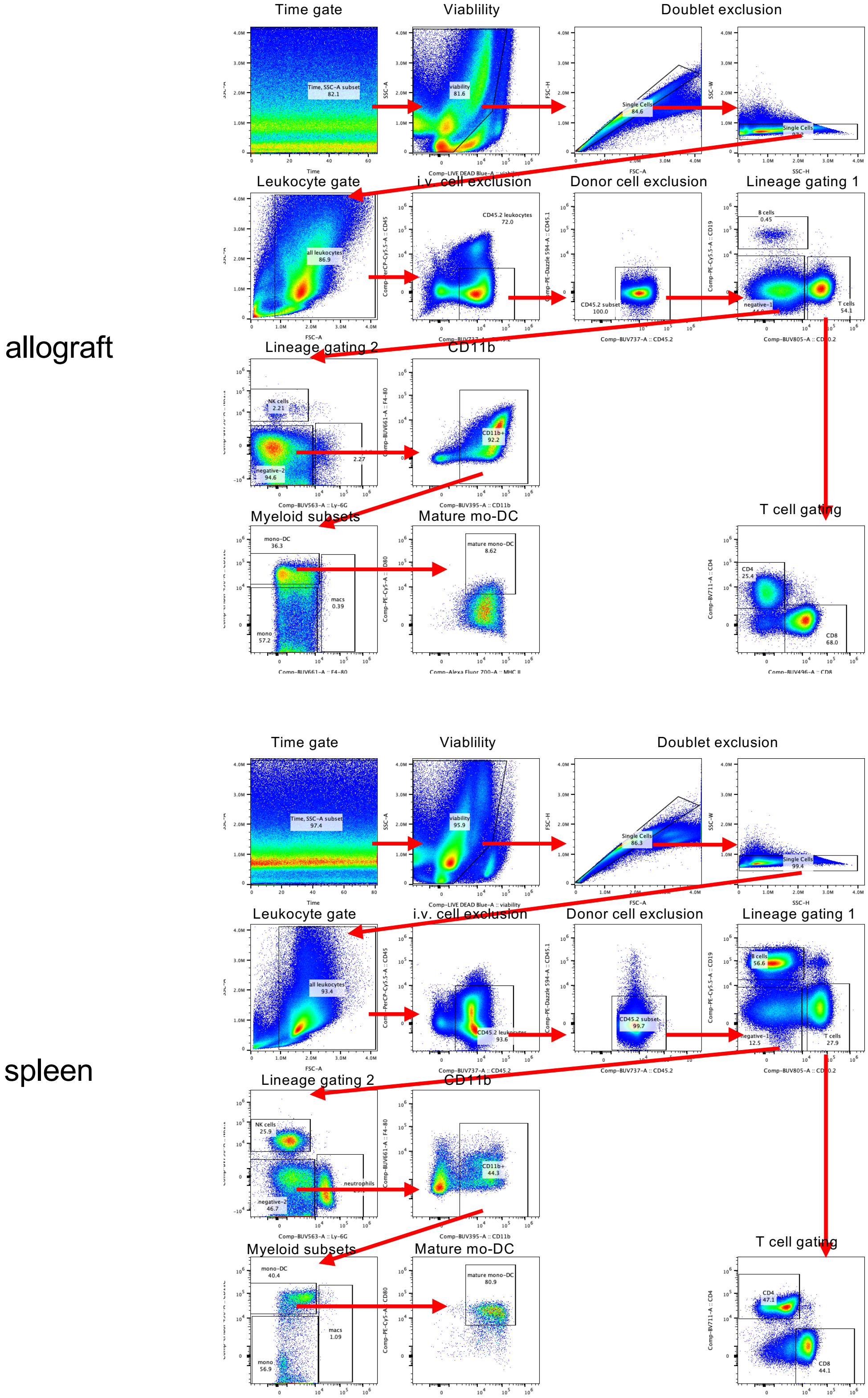


Supplemental Figure 4: **Gating strategy for identification of immune subsets in allografts and spleen.**

Representative flow cytometry gating strategy used to identify immune subsets in kidney allografts (top) and spleen (bottom) on day 7 after transplantation. Cells were gated sequentially on time, viability, single cells, leukocytes (CD45+), intravascular (i.v.) label exclusion, and donor-cell exclusion, followed by lineage gating to resolve B cells, T cells (CD4 and CD8), NK cells, neutrophils, CD11b+ myeloid cells, monocytes, mo-DC, mature mo-DC (CD80+ MHCII+), and macrophages.
